# Hydrophobic Interactions Are a Key to MDM2 Inhibition by Polyphenols as Revealed by Molecular Dynamics Simulations and MM/PBSA Free Energy Calculations

**DOI:** 10.1371/journal.pone.0149014

**Published:** 2016-02-10

**Authors:** Sharad Verma, Sonam Grover, Chetna Tyagi, Sukriti Goyal, Salma Jamal, Aditi Singh, Abhinav Grover

**Affiliations:** 1 School of Biotechnology, Jawaharlal Nehru University, New Delhi, India; 2 Kusuma School of Biological Sciences, Indian Institute of Technology Delhi, New Delhi, India; 3 Department of Bioscience and Biotechnology, Banasthali Vidyapith, Tonk, Rajasthan, India; 4 Department of Biotechnology, TERI University, Vasant Kunj, New Delhi, India; Wake Forest University, UNITED STATES

## Abstract

p53, a tumor suppressor protein, has been proven to regulate the cell cycle, apoptosis, and DNA repair to prevent malignant transformation. MDM2 regulates activity of p53 and inhibits its binding to DNA. In the present study, we elucidated the MDM2 inhibition potential of polyphenols (Apigenin, Fisetin, Galangin and Luteolin) by MD simulation and MM/PBSA free energy calculations. All polyphenols bind to hydrophobic groove of MDM2 and the binding was found to be stable throughout MD simulation. Luteolin showed the highest negative binding free energy value of -173.80 kJ/mol followed by Fisetin with value of -172.25 kJ/mol. It was found by free energy calculations, that hydrophobic interactions (vdW energy) have major contribution in binding free energy.

## Introduction

**Fig 1:**
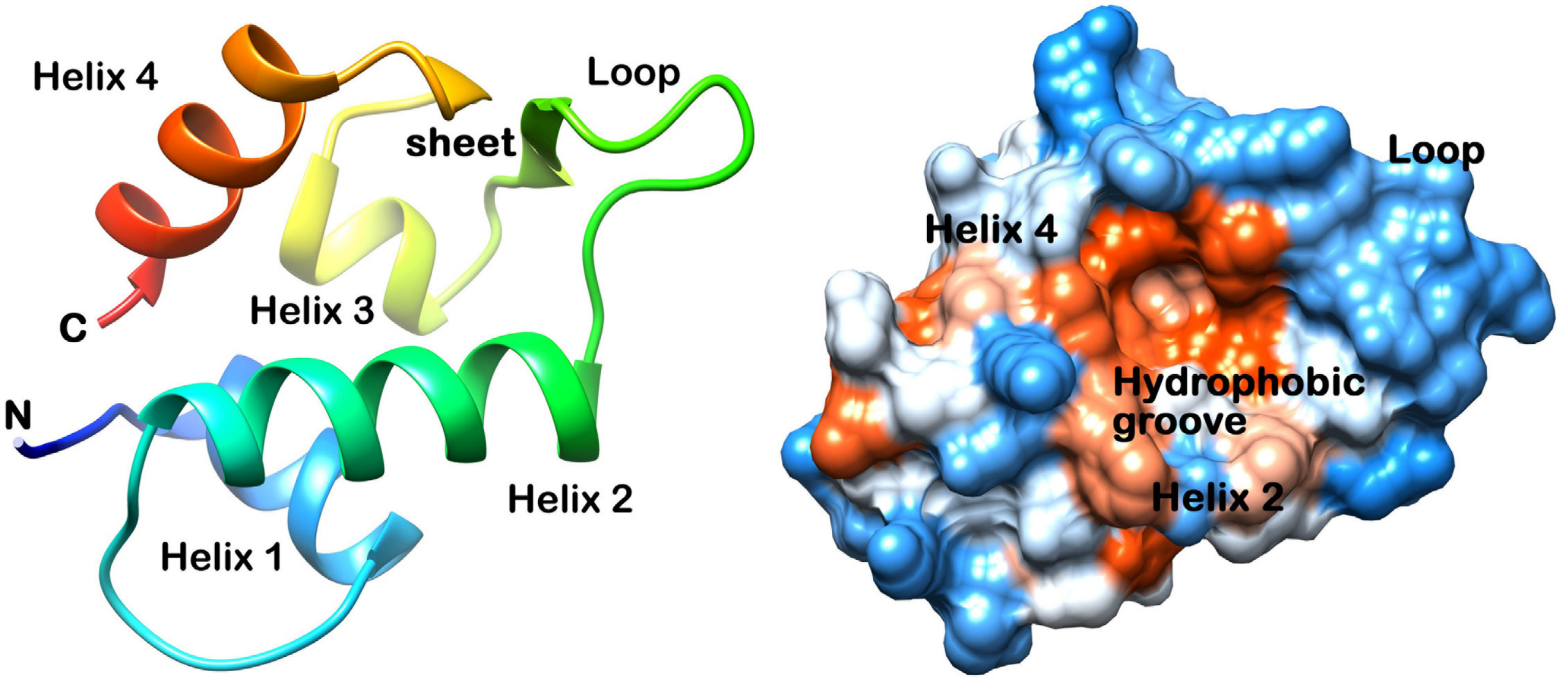
Structure of p53 binding domain of MDM2 (A) Ribbon, (B) Surface.

**Fig 2:**
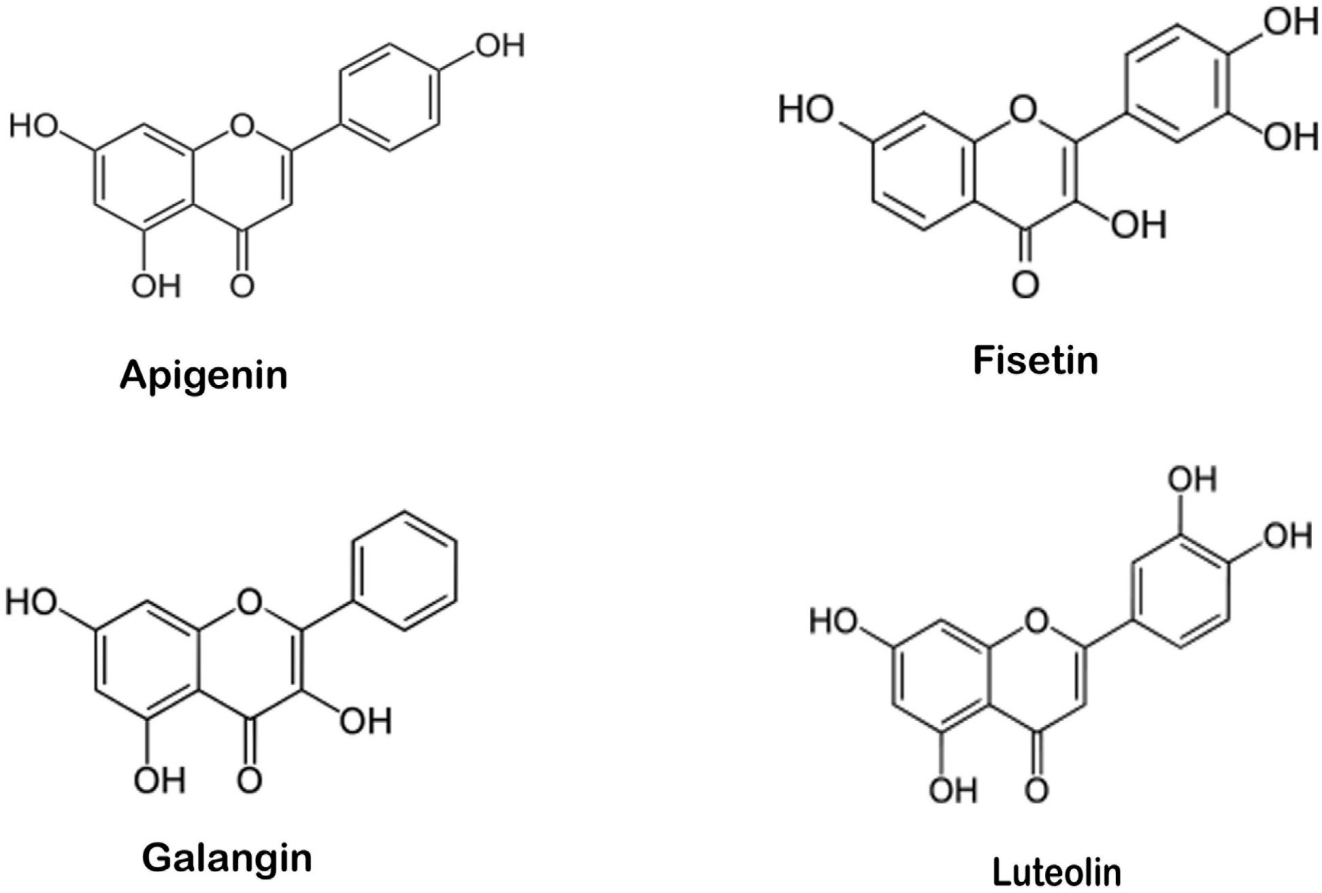
Structure of polyphenols.

p53, a tumor suppressor protein, has critical role in regulation of the cell cycle, apoptosis, and DNA repair to prevent malignant transformation [1–3]. Over expressed MDM2 regulates activity of p53 and inhibits its binding with DNA in tumors [4]. Several peptide inhibitors that mimic p53 have been reported but they exhibit modest effects because they have poor membrane permeability [5–9]. The structure of MDM2 (p53 binding region) consists a hydrophobic groove like structure formed by two helices and a loop. Two sheet structures form the back of groove (Fig 1). The important residues Leu54, Leu57, Gly58, Ile61, Met62, Tyr67, Gln72, His73, Val75, Phe91, Val93, His96, Ile99 and Tyr100 are together known as the structural component which interacts with p53 residues [10, 11]. Naturally occurring polyphenolic phytochemicals have been reported to inhibit cancer [12, 13] and also show potential binding to MDM2 in its hydrophobic grooves [14, 15]. In this study, we tried to elucidate the binding of polyphenols (Apigenin, Fisetin, Galangin and Luteolin) (Fig 2) to MDM2 groove with the help of molecular docking and molecular dynamic simulation along with MM/PBSA free energy calculations.

Apigenin is widely found in many fruits and vegetables, including chamomile, parsley, onions, tea, orange and wheat sprouts [16]. Fisetin is present in strawberries, apples, persimmons, kiwis, cucumbers and onions [17]. Galangin is a found in *Alpinia officinarum* (lesser galangal) [18]. Luteolin concentrations are found in peanut hulls and in *Reseda luteola L*., the Dyer's weld are high as compared to food [19, 20].

## Materials and Methods

### Molecular Docking of natural polyphenols at the MDM2 groove

Molecular-docking was performed using molecular docking suite AutoDock 4.0. The crystal structure of MDM2 (PDB ID 1RV1) was obtained from the RCSB protein data bank. The structures of polyphenols (Apigenin, Fisetin, Galangin and Luteolin) were generated from SMILE strings. All the heteroatoms were removed during the preparation of protein coordinate file. All the missing atoms were repaired by AutoDock. Adaptive local search based Lamarckian genetic algorithm [21] was used as a search parameter. Short range van der Waals and electrostatic interactions, hydrogen bonding, entropy losses were included for energy-based AutoDock scoring function [21, 22]. In the study, the Lamarckian GA parameters used were the number of runs, 20; population size, 150; the maximum number of eval, 250,00,000; the number of generations, 27,000; rate of gene mutation, 0.02; and the rate of cross over, 0.8. Blind docking is carried out using grid size of 126 × 126 × 126 along the X, Y, and Z axes with 0.375 Å spacing. RMS cluster tolerance was set to 2.0 Å.

### Molecular dynamic simulation

MD simulation of the complex was carried out using the GROMOS96 43a1 force field [23, 24] of the GROMACS 4.5.4 package. Autodock generated lowest binding energy (most negative) docking conformation of MDM2–polyphenol complex which was taken as the initial conformation for MD simulation. The topology parameters of proteins were created by means of the Gromacs program. The topology parameters of taxifolin were built using the Dundee PRODRG server [25]. The complex was immersed in a cubic box of extended simple point charge water molecules [26, 27]. Energy minimization was performed using the steepest descent method of 10,000 steps followed by the conjugate gradient method for 10,000 steps, to release conflicting contacts. Position-restrained dynamics simulation (equilibration phase) (NVT and NPT) of the system was done at 300 K for 200 ps followed by MD production run for 15 ns. For the purpose of analysis, the atomic coordinates were recorded every 1.0 ps during the MD simulation. All the structural images were generated using Chimera [28].

### Binding free energy calculations

The molecular mechanics Poisson Boltzmann surface area (MM/PBSA) method [29] is the widely used method for binding free energy calculations from the snapshots of MD trajectory. The binding free energies of the complexes between polyphenols and MDM2 were analyzed during equilibrium phase by taking snapshots at an interval of 1.5 ps from 13 to 15 ns MD simulations, using g_mmpbsa tool of Gromacs [30].

Particularly, the binding free energy of ligand-protein complex in solvent was expressed as:

$$\Delta {\text{G}}_{\text{binding}}\;=\;{\text{G}}_{\text{complex}}\;-\;({\text{G}}_{\text{protein}}\;+\;{\text{G}}_{\text{ligand}})$$

where G_complex_ is the total free energy of the protein-ligand complex, G_protein_ and G_ligand_ are total energy of separated protein and ligand in solvent, respectively. The free energy for each individual G_complex_, G_protein_ and G_ligand_ were estimated by:

$${\text{G}}_{\text{x}}\;=\;{\text{E}}_{\text{MM}}\;+\;{\text{G}}_{\text{solvation}}$$

where x is the protein, ligand, or complex. E_MM_ is the average molecular mechanics potential energy in vacuum and G_solvation_ is free energy of solvation. The molecular mechanics potential energy was calculated in vacuum as following:

$${\text{E}}_{\text{MM}}\;=\;{\text{E}}_{\text{bonded}}\;+\;{\text{E}}_{\text{non}-\text{bonded}}\;=\;{\text{E}}_{\text{bonded}}\;+\;({\text{E}}_{\text{vdw}}\;+\;{\text{E}}_{\text{elec}})$$

where E_bonded_ is bonded interaction including of bond, angle, dihedral and improper interactions and E_non-bonded_ is non-bonded interactions consisting of van der Waals (E_vdw_) and electrostatic (E_elec_) interactions. ΔE_bonded_ is always taken as zero [31].

The solvation free energy (G_solvation_) was estimated as the sum of electrostatic solvation free energy (G_polar_) and apolar solvation free energy (G_non-polar_):

$${\text{G}}_{\text{solvation}}\;=\;{\text{G}}_{\text{polar}}\;+\;{\text{G}}_{\text{non}-\text{polar}}$$

where G_polar_ was computed using the Poisson-Boltzmann (PB) equation [29] and G_non-polar_ estimated from the solvent-accessible surface area (SASA) as equation following:

$${\text{G}}_{\text{non}-\text{polar}}\;=\;\gamma \text{SASA}\;+\;\text{b}$$

where γ is a coefficient related to surface tension of the solvent and b is fitting parameter. The values of the contats are as follows:

γ = 0.02267 Kj/Mol/Å^2^ or 0.0054 Kcal/Mol/Å^2^

b = 3.849 Kj/Mol or 0.916 Kcal/Mol

### Principal component analysis (PCA)

PCA was performed to obtain a mass-weighted covariance matrix of the protein atom displacement which is indicative of dominant and collective modes of the protein from the overall dynamics of the MD trajectory. This covariance matrix is diagonalized to extract a set of eigenvectors and eigenvalues that reflect concerted motion of the molecule [32–35]. The Gromacs in-built tool g_covar was used to yield the eigenvalues and eigenvectors by calculating and diagonalizing the covariance matrix, whereas the g_anaeig tool was used to analyze and plot the eigenvectors [34].

## Results and Discussion

**Fig 3:**
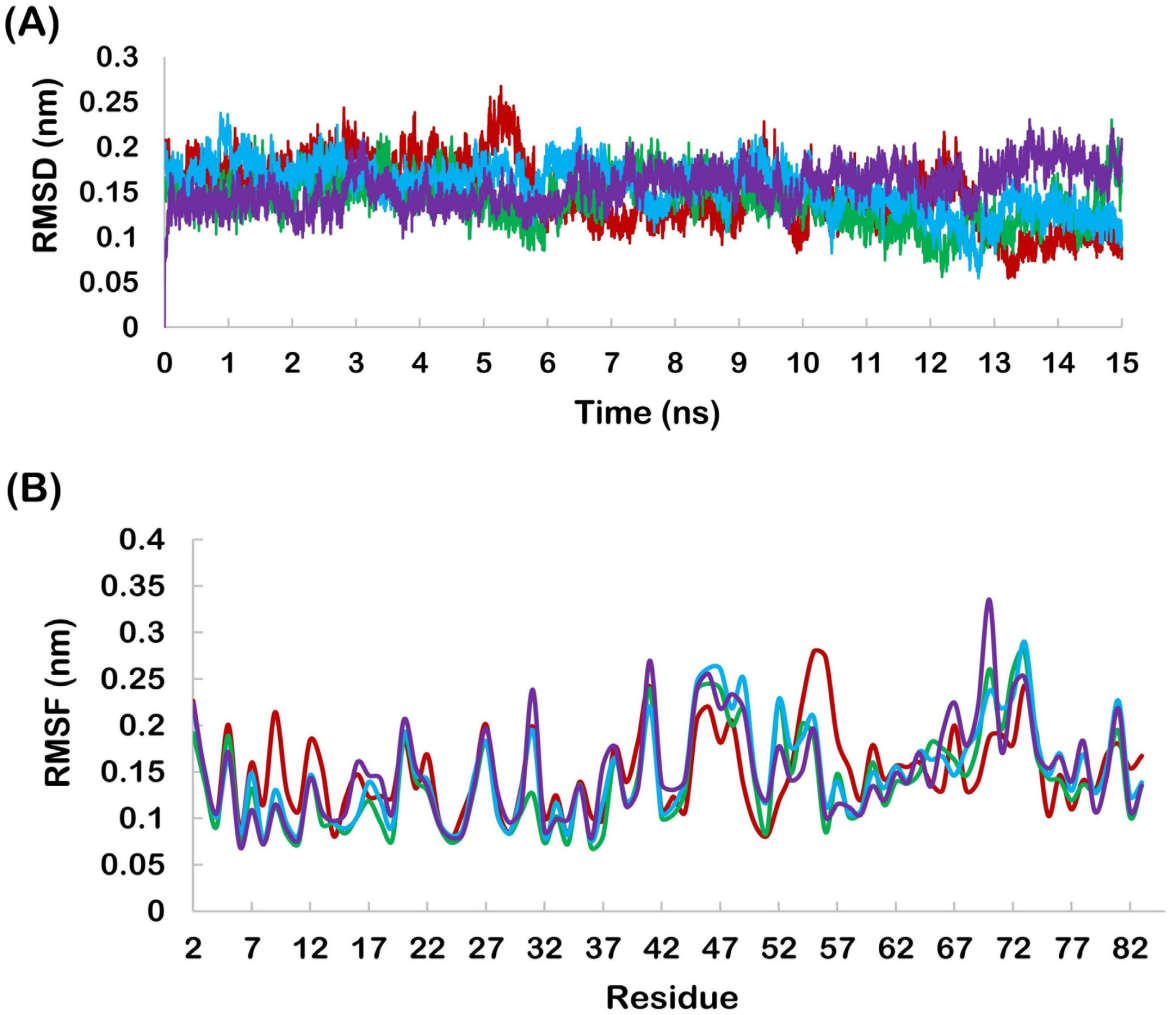
(A) backbone RMSD of MDM2 (Red-apigenin, Green- fisetin, Blue-galangin, Purple-luteolin), (B) RMSF profile of MDM2 (Red-apigenin, Green- fisetin, Blue-galangin, Purple-luteolin).

Molecular docking results revealed that Apigenin, Fisetin, Galangin and Luteolin bind to hydrophobic groove of MDM2 with lowest binding energy values of (most negative) -5.62, -6.02, -5.78 and -6.39 kcal/mol respectively. These docking conformations of MDM2-polyhenol complexes, generated by Autodock, were taken as initial conformation for MD simulation. Fig 3A shows that the RMSD profiles were always less than 0.25 nm for all ligands bound to MDM2 backbone during the entire simulation suggesting the suitability of MD simulation run. Fig 3B shows the RMSF profile of MDM2. In all the complexes, MDM2 showed fluctuation within the range of 0.30 nm in a very similar pattern.

**Fig 4:**
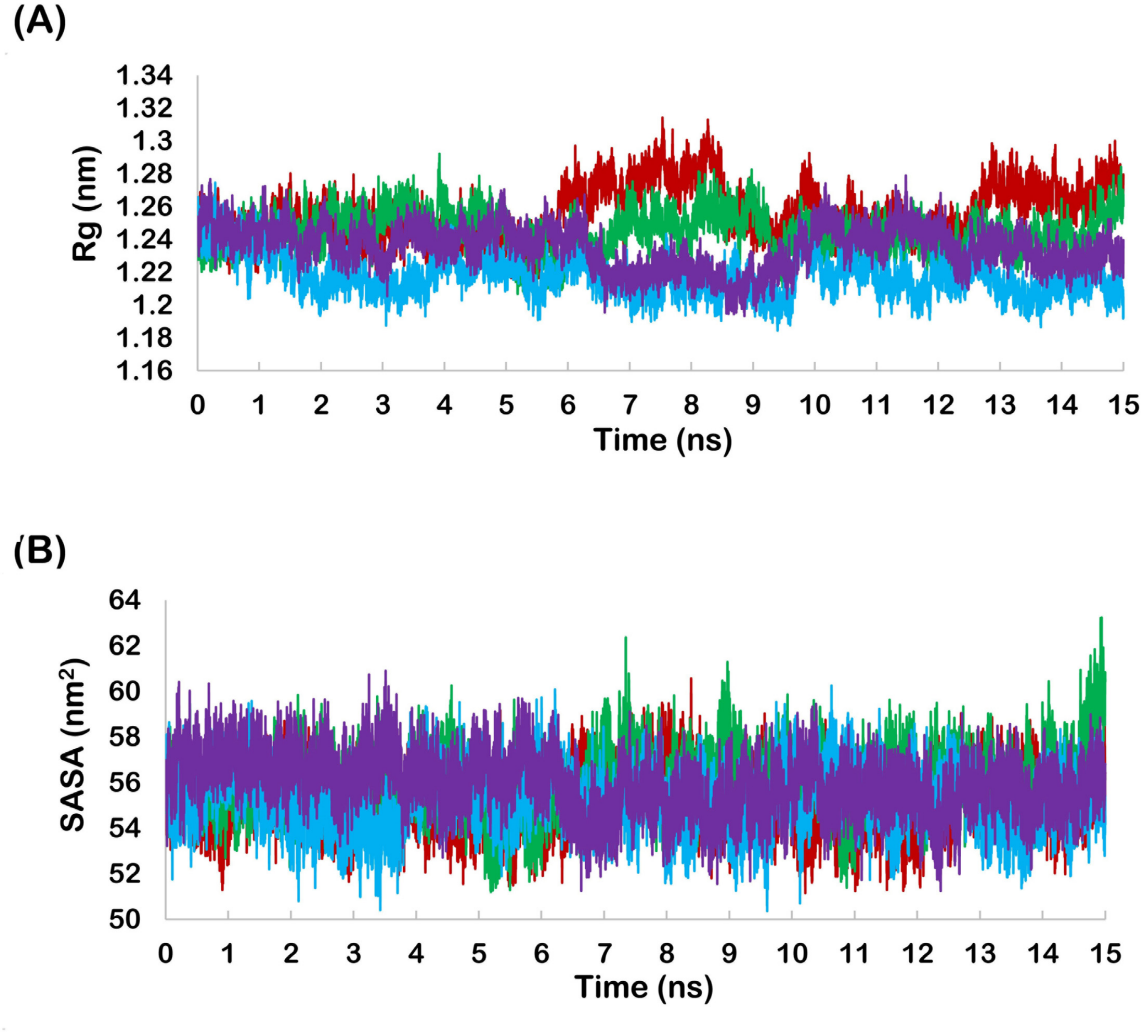
(A) Radius of gyration profile of MDM2 (Red-apigenin, Green- fisetin, Blue-galangin, Purple-luteolin)., (B) SASA profile of MDM2 (Red-apigenin, Green- fisetin, Blue-galangin, Purple-luteolin).

The root mean square distance between center of gravity and ends of an object denotes radius of gyration (Rg). The radius of gyration provides indication about the level of compaction in the protein structure. Apigenin and Fisetin bound MDM2 showed slightly increased radius of gyration while Galangin and Luteolin bound MDM2 showed slightly decreased radius of gyration (Fig 4A). Solvent accessible surface area (SASA) indicates the solvent exposed surface of protein and hence the folding of exposed part of proteins. Galangin bound MDM2 showed slightly increased SASA while other polyphenol bound MDM2 showed slightly decreased SASA (Fig 4B).

**Fig 5:**
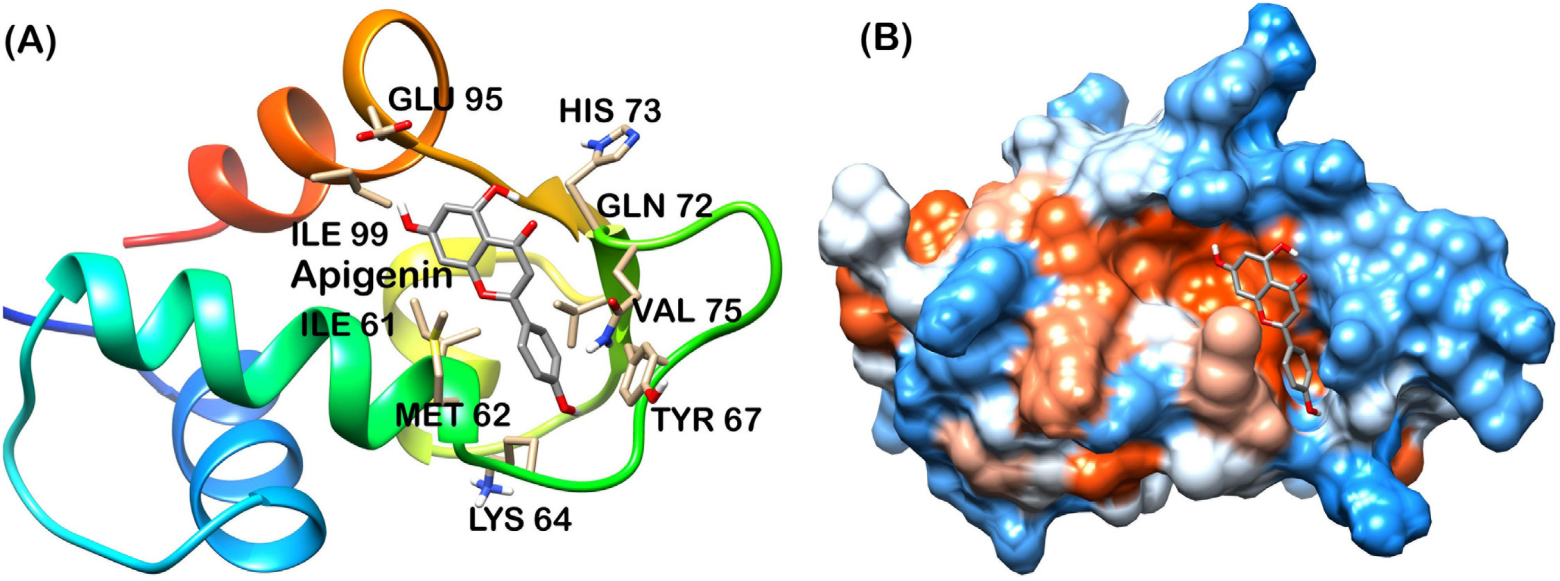
Apigenin-MDM2 complex at 15 ns (A) cartoon representation, (B) surface structure.

**Fig 6:**
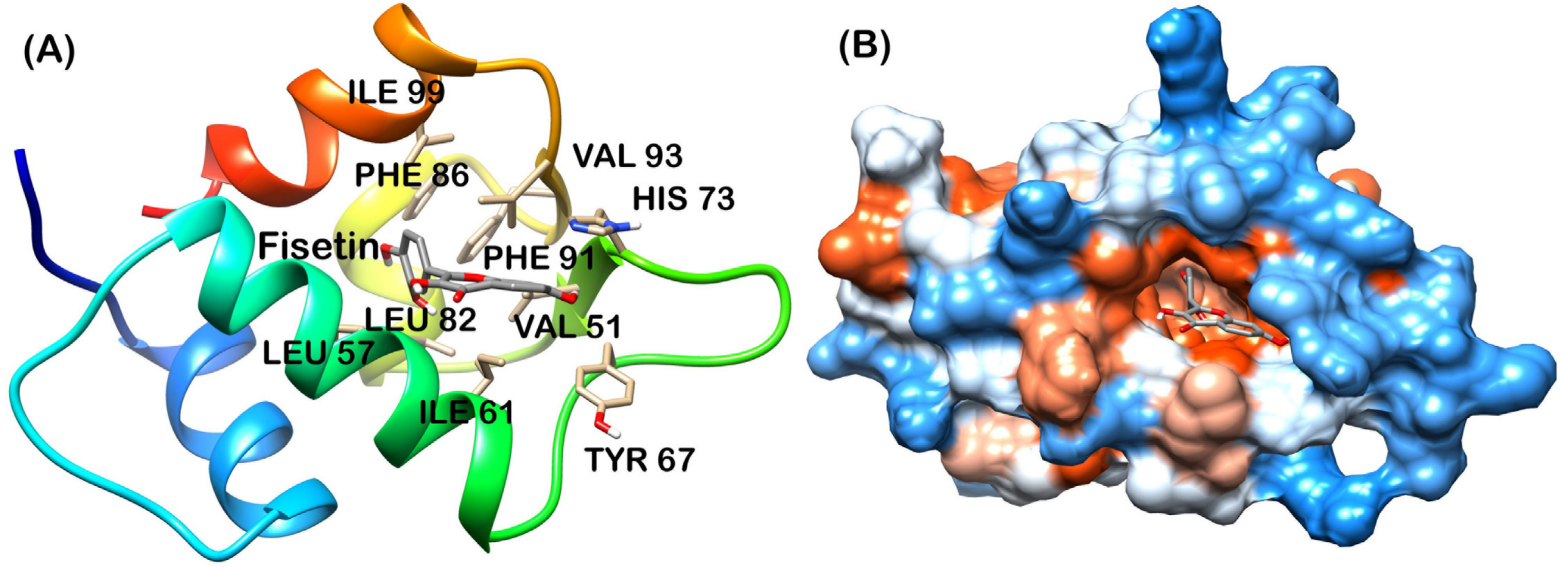
Fisetin-MDM2 complex at 15 ns (A) cartoon representation, (B) surface structure.

**Fig 7:**
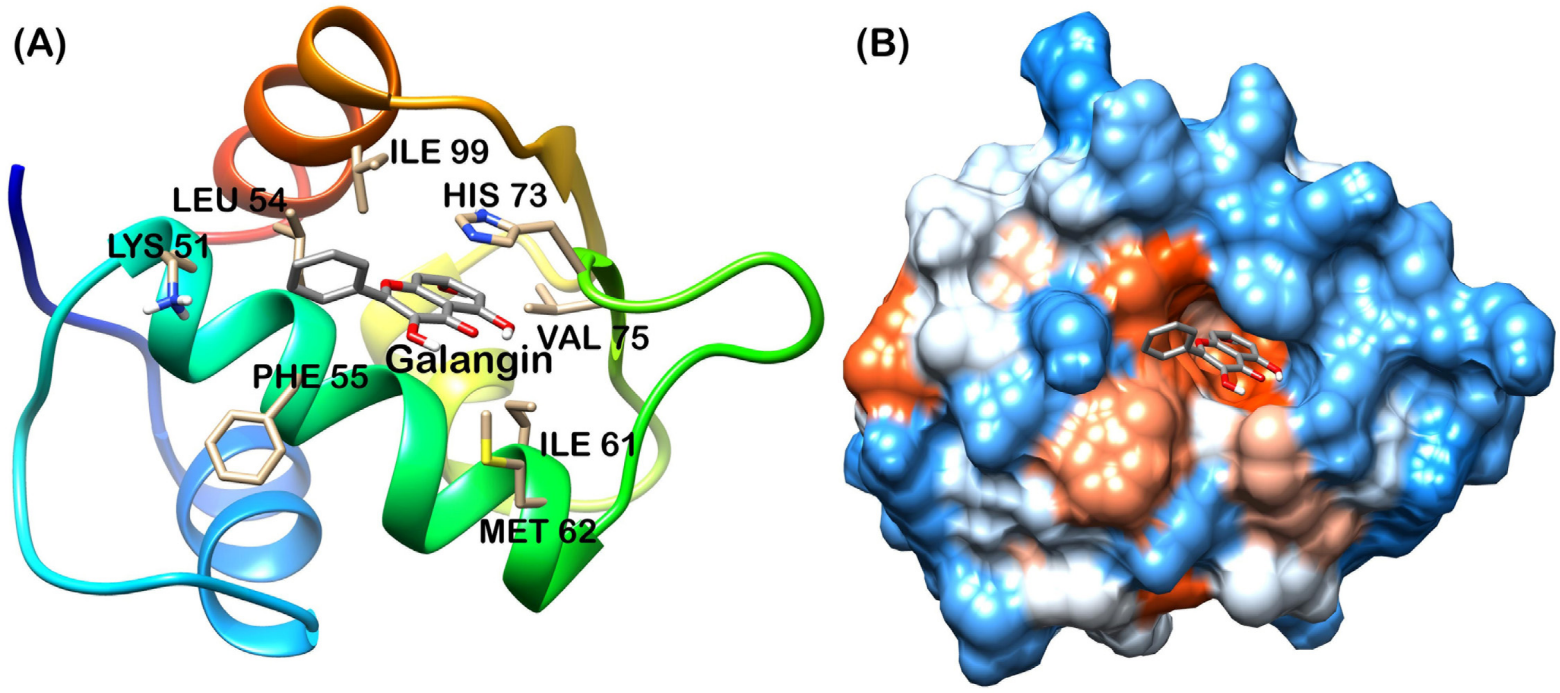
Galangin-MDM2 complex at 15 ns (A) cartoon representation, (B) surface structure.

**Fig 8:**
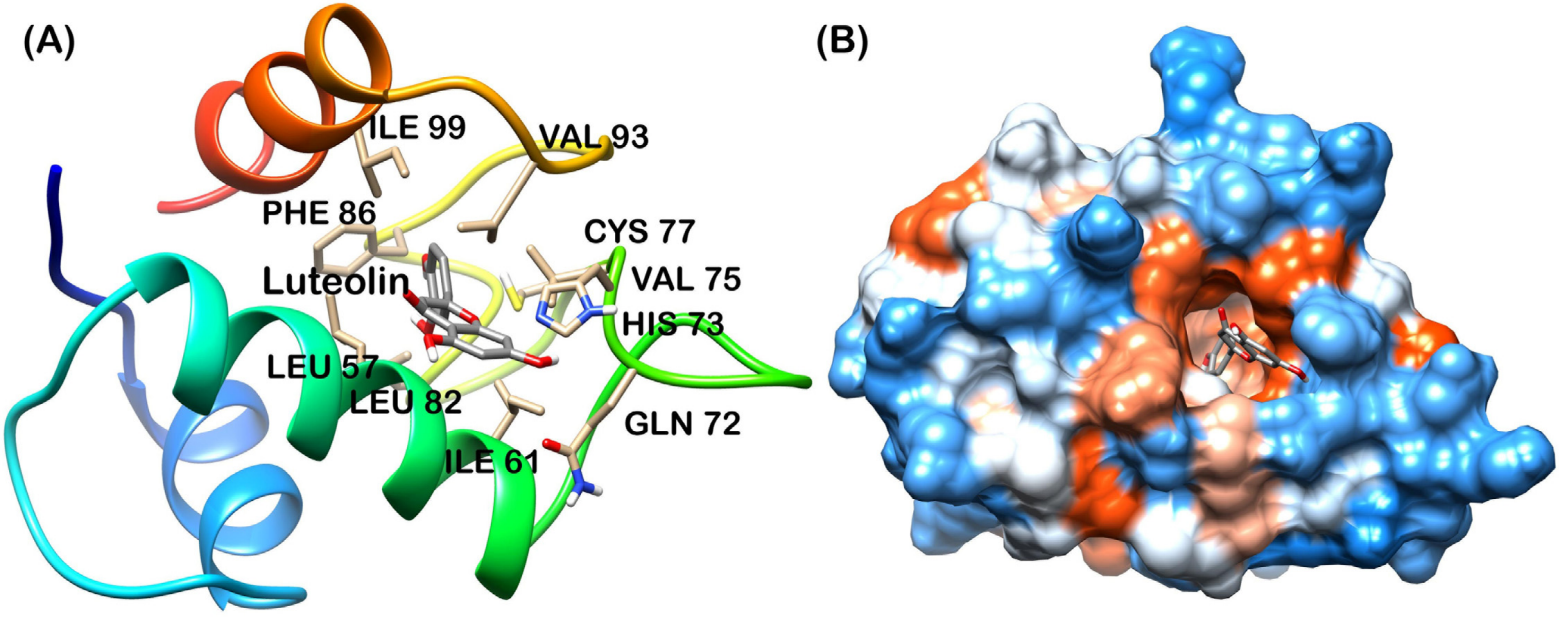
Luteolin-MDM2 complex at 15 ns (A) cartoon representation, (B) surface structure.

Analysis of final pose of MDM2-ligand complex after 15 ns molecular dynamics simulation revealed that polyphenols stably bound to the hydrophobic groove. Fig 5A shows Apigenin surrounded by the MDM2 hydrophobic groove residues. Comparative surface analysis of final and initial structures revealed significant conformational changes. Residues were found to overlap with bound Apigenin (Fig 5B). Even as the binding site for all the polyphenols is same, yet Fisetin, Galangin and Luteolin brought more significant change in the hydrophobic groove and were found to be deeply penetrated in the groove as shown in Figs 6, 7 and 8 respectively. These polyphenols induced deep pore-like structure and could accommodate in it.

**Fig 9:**
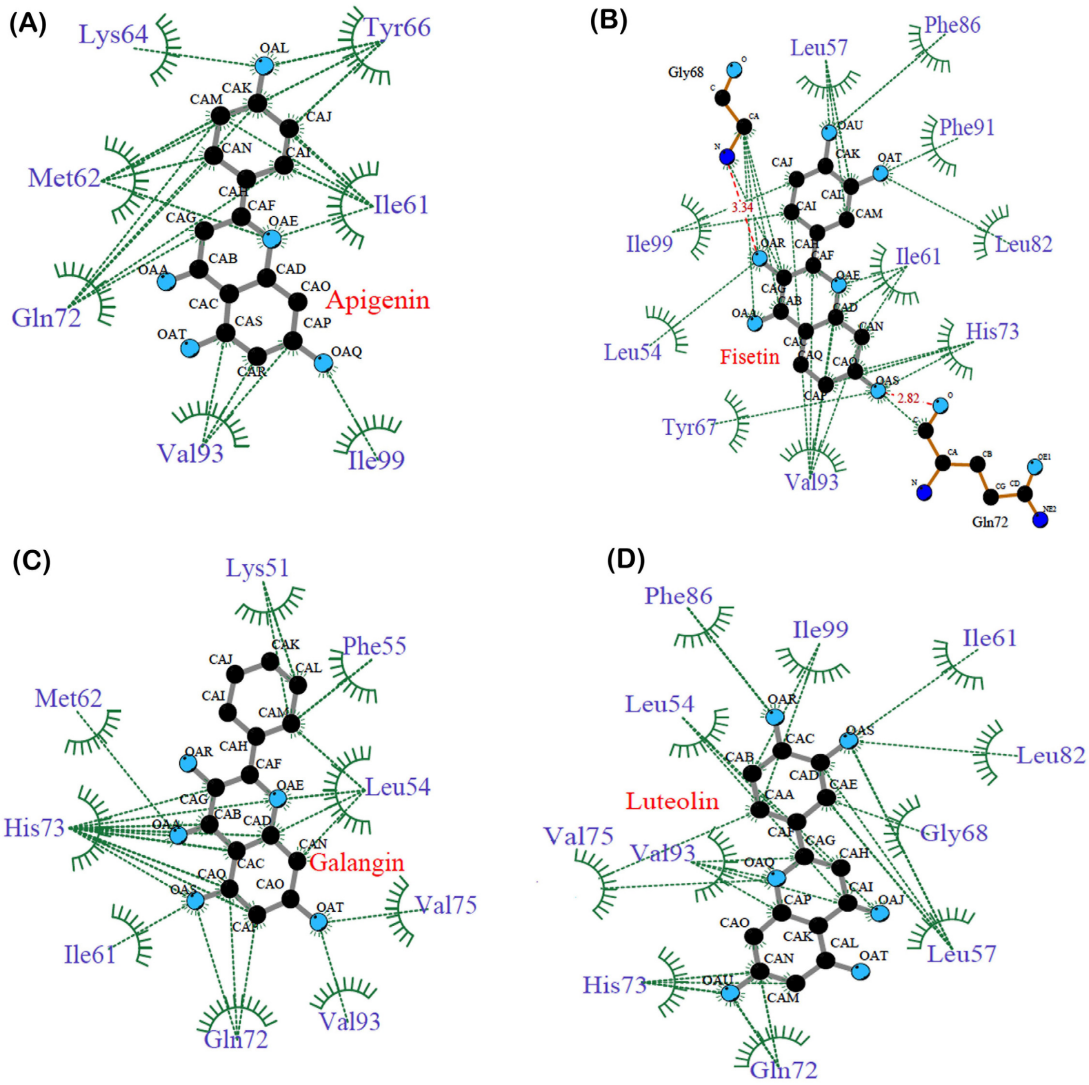
Polyphenol-MDM2 interaction plot generated by Ligplot (A) Apigenin, (B) Fisetin, (C) Galangin, (D) Luteolin.

The interaction plot, generated by Ligplot [36] showed massive hydrophobic interaction between residues and polyphenols. The interacting residues include Ile61, Met62, His73, Tyr67, Leu58, Phe86, Val93 and Ile99 (Fig 9). These residues have been known to interact with p53 residues through previous studies [10, 11].

### Free energy calculations

Molecular dynamic simulations were used to calculate binding free energy using MM/PBSA method. Snapshots were extracted at every 15 ps of stable intervals from 13–15 ns MD trajectory. The binding free energy and its corresponding components obtained from the MM/PBSA calculation of the MDM2-polyphenol complexes are listed in Table 1. The results indicated that Luteolin possessed highest negative binding free energy value of -173.80 kJ/mol followed by Fisetin with value of -172.25 kJ/mol. Apigenin and Galangin showed affinity with value of -139.48 and -142.48 kJ/mol, respectively.

**Table 1:** Average MM/PBSA free energies of MDM2-polyphenol complexes calculated from the MD simulations performed in triplicate.

Ligands	Van der waal (kJ/mol)	Electrostattic (kJ/mol)	Polar salvation (kJ/mol)	Non-polar salvation (kJ/mol)	Binding energy (kJ/mol)
**Apigenin**	-149.81±11.91	-23.65±1.41	47.08±10.91	-14.22±0.76	-139.48±3.53
**Fisetin**	-154.62±1.20	-36.39±13.53	32.29±0.23	-13.74±0.50	-172.25±13.70
**Galangin**	-152.04±13.27	-12.98±5.90	36.54±4.60	-13.01±0.93	-142.48±1.40
**Luteolin**	-170.40±20.29	-12.42±9.10	23.84±0.97	-14.82±0.028	-173.80±4.34

Moreover, van der Waals and electrostatic interactions and non-polar solvation energy negatively contribute to the total interaction energy while only polar solvation energy positively contributes to total free binding energy. In terms of negative contribution, van der Waals interaction gives much larger contribution than electrostatic interactions for all the cases. The non-polar free energy contributes relatively less as compared to the total binding energy. This indicates that non-polar solvation energy, van der Waals and electrostatic interaction together contribute to the MDM2-polyphenol complex stability. From the data collected by mm/pbsa calculations and the interaction plot generated by ligplot, it is seen that hydrophobic interactions are dominant in all complexes. High negative value of vdW energy represents the massive hydrophobic interaction between MDM2 and polyphenols.

**Fig 10:**
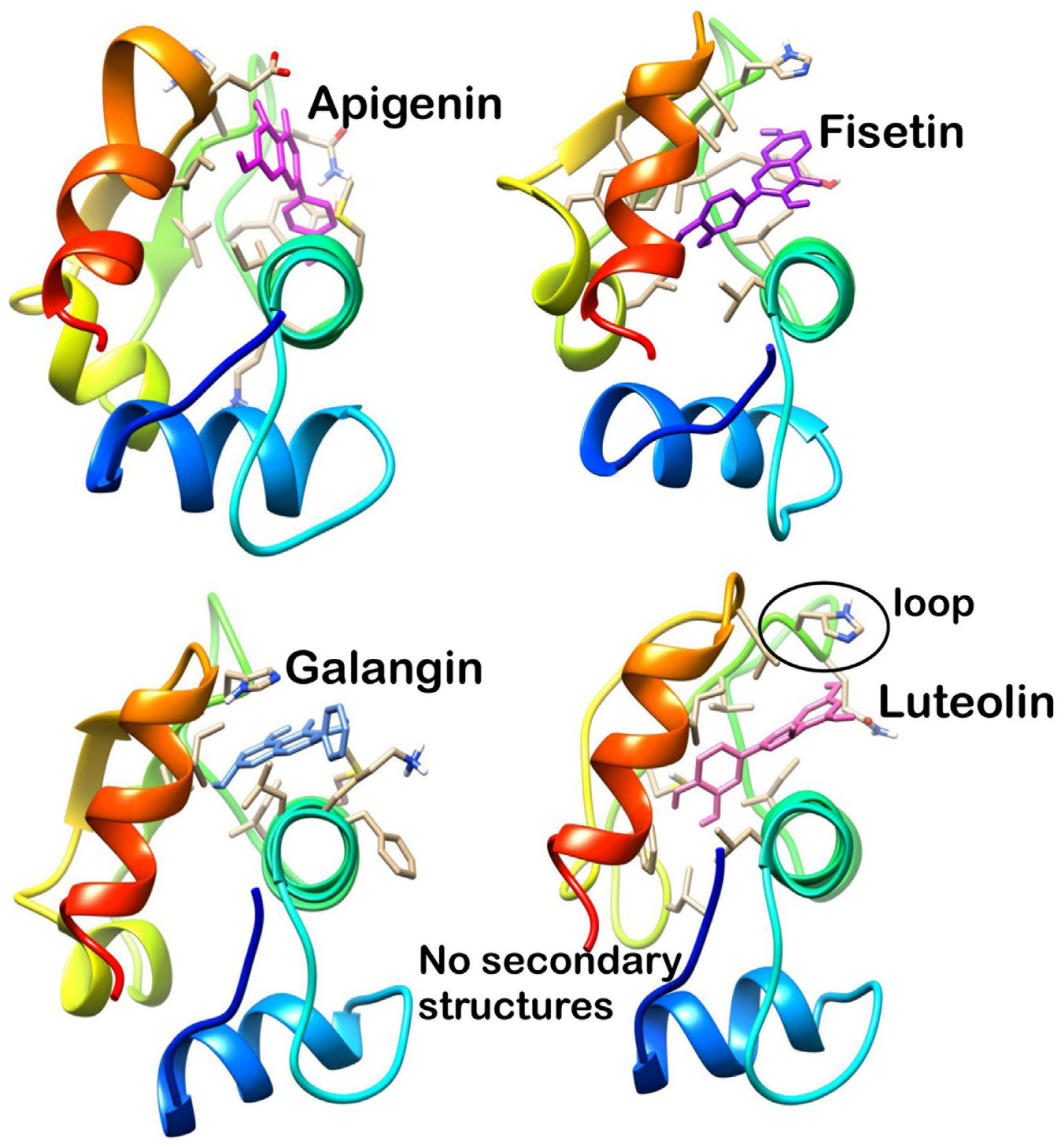
Penetration of polyphenol in hydrophobic groove of MDM2.

Analysis of final snapshot at 15 ns revealed that Luteolin and Fisetin deeply penetrate into the hydrophobic groove. The loop (residue 41 to 49) was found to be in closer proximity in Luteolin and Fisetin bound MDM2 (Fig 10). These can be possible reasons responsible for the higher hydrophobic interaction with Luteolin and Fisetin and possible reason of higher binding energy. The sheet structures were absent in the Luteolin bound form which may confer the flexibility of groove and facilitate the penetration of Luteolin.

### Principal component analysis (PCA)

**Fig 11:**
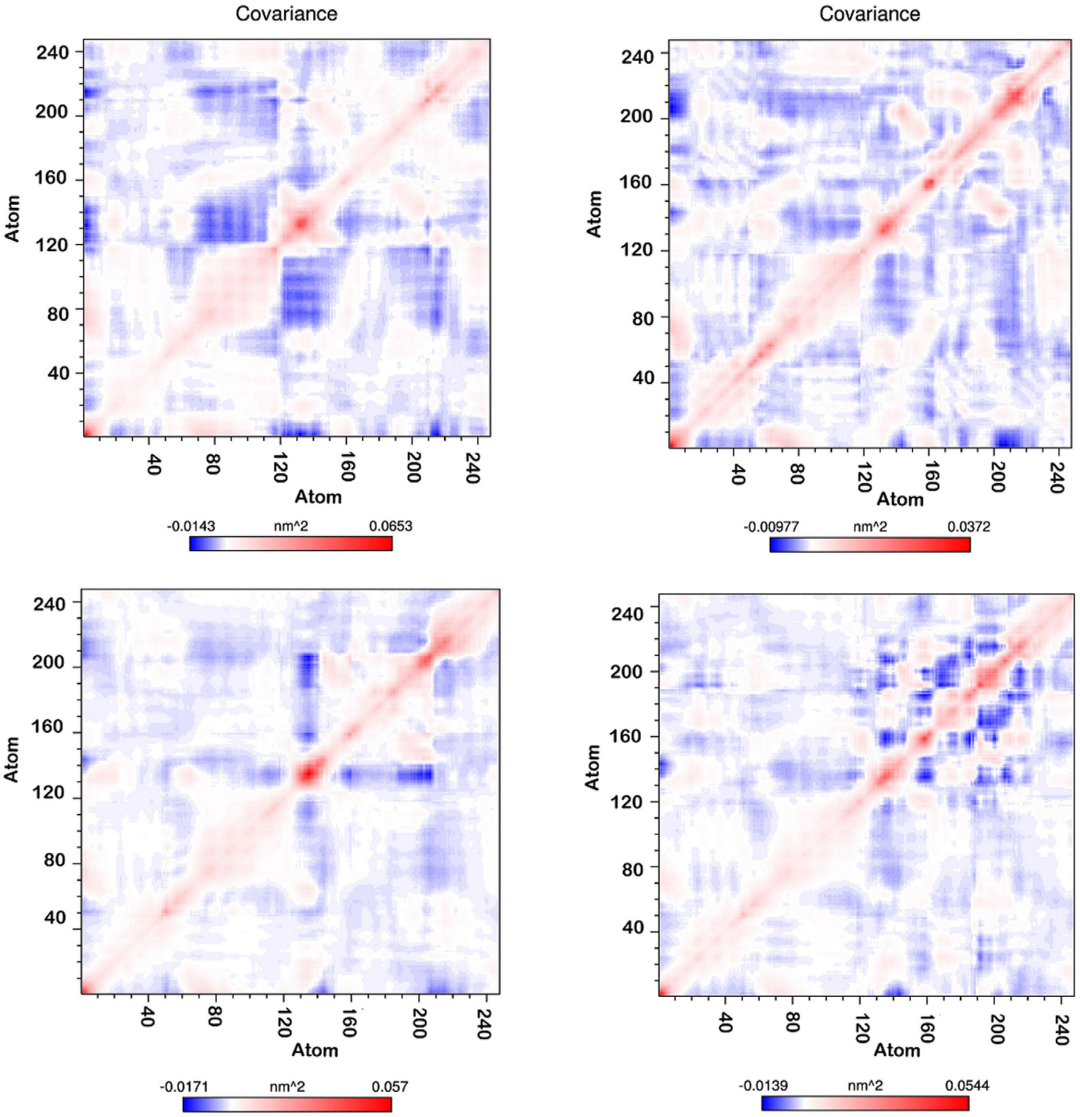
Covariance matrix of MDM2 during 15 ns MD simulation (Upper left-Apigenin, Upper right-Fisetin, Lower left-Galangin, Lower right-Luteolin).

The MD trajectory of system was inspected with the principal components to better understand the conformational changes of MDM2 with all polyphenol bound forms. Correlated motion plot shows how atoms move relative to each other. Motions can be positively correlated (in the same direction), anti-correlated (in the opposite direction), or uncorrelated [37]. The positive and negative limits are shown in Fig 11. Anti-correlated motions were dominant in all the forms. Summary of significant motions is presented in Table 2. These motions collectively drive the structure of hydrophobic groove and also the intensity of hydrophobic interaction. Difference in final hydrophobic groove structure of MDM2 forms can be observed in Figs 5B to 8B.

**Table 2:** Dominant motions of atom in helix2, helix 4 and loop.

	Helix 2-Helix 4	Helix 2-Loop	Loop-Helix 4
**Apigenin**	Anti-correlated, anticorrelated with helix 4 terminal residues	Strongly anticorrelated	Mixed (anticorrelated and correlated)
**Fisetin**	Anti-correlated, anticorrelated with helix 4 terminal residues	Anticorrelated	Mixed (anticorrelated and correlated)
**Galangin**	Anti-correlated, uncorrelated with helix 4 terminal residues	Anticorrelated	Mixed (anticorrelated and uncorrelated)
**Lueolin**	Anti-correlated, uncorrelated with helix 4 terminal residues	Weakly anticorrelated	uncorrelated

Nutlin-3 is a well-known inhibitor of p53-MDM2 interaction and activates p53 in cancer cells [1, 38–40]. Nutlin-3a, which is an active emantiomer of Nutlin-3, is an MDM2 antagonist and found to be very effective in the treatment of Ewing’s sarcoma cells [41–43]. Nutlin-3a and polyphenols shared similar binding site on MDM2. The binding energy value for MDM2-Nutlin-3a complex was -9.11 kcal/Mol [44] calculated by MM/PBSA method which is very high as compared to polyphenols. However in case of nutlin binding energy found to be -43.5 kcal/Mol (entropic term is not included) which is slightly lower than polyphenols. Same study noted that the MDM2-nutlin interaction driven by the van der Waals interactions as in case of polyphenols [45]. This comparison revealed that polyphenols (Apigenin, Fisetin, Galangin and Luteolin) have tremendous potential to act as the MDM2 inhibitors.

## Conclusions

All polyphenols (Apigenin, Fisetin, Galangin and Luteolin) bind to hydrophobic groove of MDM2 and the binding was found to be stable throughout MD simulation. Luteolin binds with highest negative binding energy value and shows highest potency towards MDM2 inhibition. Although, the MDM2 residues interacting with polyphenols were the same for all but, the change in groove structure significantly affected the position of polyphenol in the groove and also its binding energy. The hydrophobic interactions were solely responsible for stable complex formation as revealed by the vdW energy and ligplot analysis. Finally, on the basis of data obtained during the study, it can be concluded that these polyphenols have the potential to be used as lead molecules for the inhibition of MDM2 with Luteolin being the top candidate. This approach can be used to screen out a huge number of natural compounds for their potency in anti-cancer treatment.
